# On optimal liver T2* measurement: region of interest or pixel-wise?

**DOI:** 10.1186/1532-429X-14-S1-P293

**Published:** 2012-02-01

**Authors:** Yanqiu Feng, Peter D Gatehouse, Peiguo Liu, John-Paul Carpenter, Dudley J Pennell, David N Firmin, Wufan Chen, Taigang He

**Affiliations:** 1 School of Biomedical Engineering, Southern Medical University, Guangzhou, China; 2 CMR Unit, Royal Brompton Hospital, London, UK; 3 National Heart and Lung Institute, Imperial College, London, UK; 4 School of Electronic Science and Engineering, National University of Defence Technology, Changsha, China

## Background

MRI T2* measurement is playing an important role in liver iron quantification. There are currently two clinical approaches measuring T2* in the region of interest ( ROI): the first is to fit the means of signals in the ROI (mean-ROI fitting, MRF) and the second to average the pixel-wisely fitted (PWF) T2*s in the ROI. In a recent study, Marro et al [1] compared the performances of PWF and MRF through mathematical simulations but only the offset model was evaluated.

The purpose is to determines the optimal liver T2* measurement by evaluating the performances of MRF and PWF with 4 different fitting models on simulation and phantom data.

## Methods

For numerical simulations, free induction decay (FID) signals were synthesized. T2* = [0.67, 0.8, 1.0, 1.25, 1.67, 2.0, 2.5, 3.33, 5, 6.67, 10, 20 ms]; 16 TEs from 0.97ms to 14 ms equally spaced. Signal to noise ratio was defined as SNR=s_0/σ , where s_0 denotes the signal at TE=0 and σ denotes the standard deviation of noise. The Rician noise was added to the generated FIDs. The means of the measured T2*s of 200 independent simulations were calculated to evaluate the accuracies.

A dedicated T2* phantom (containing 12 tubes with similar T2* as listed above) was scanned with a GRE Sequence: FA = 5°, TR = 200 ms. TEs were selected the same as those from the simulation study. The numbers of average were set as 1 and 128 to acquire images of low and high SNR respectively.

MRF and PWF together with the truncation [2], the offset [2], the SQEXP, and the NCEXP [3] models were implemented to calculate average T2*s in the ROIs. T2*s estimated from the 128-average images, assumed to be noise-free, were used as the reference for evaluating the accuracy of T2* measurements made from low SNR data.

## Results

As shown in Figure 1, the measurement errors of MRF are less than those for PWF for the truncation, SQEXP and NCEXP models, especially at low T2* values and low SNR. For the offset model, no significant differences can be observed between MRF and PWF when T2*s are small except that PWF produces a large overestimate error at T2* = 0.67 ms when SNR = 15.

As shown in Figure 2 (right), when SNR is low, MRF consistently produces more accurate T2* than PWF for all the four models at low T2*s. At T2* = 0.55 ms, PWF fitting with the truncation and offset models overestimates T2* significantly.

## Conclusions

Results from the simulation and phantom studies demonstrate that MRF outperforms PWF for all the four fitting models when the noise is the predominant source of errors. For very short T2* values, the PWF method using the truncation and offset models overestimate T2* values, which may lead to clinical misdiagnosis. Future work is therefore needed to improve the accuracy of the pixel-wise relaxation mapping before it can be used as a relible tool in clinical practice.

## Funding

This study was supported by the UK NIHR Cardiovascular Biomedical Research Unit of Royal Brompton Hospital and Imperial College, London. Dr Feng is supported by the National Basic Research Program of China (973 Program) (Grant No. 2010CB732502) and National Natural Science Funds of China (Grant No.30800254). Dr He was supported by Wellcome Trust Value In People (VIP) award and now holds a British Heart Foundation (BHF) Intermediate Basic Science Fellowship (FS/08/26225).
